# TGFβ2 is a Prognostic Biomarker for Gastric Cancer and is Associated With Methylation and Immunotherapy Responses

**DOI:** 10.3389/fgene.2022.808041

**Published:** 2022-05-10

**Authors:** Bangling Han, Tianyi Fang, Yimin Wang, Yongle Zhang, Yingwei Xue

**Affiliations:** Department of Gastrointestinal Surgery, Harbin Medical University Cancer Hospital, Harbin, China

**Keywords:** gastric cancer, TGFβ2, NDA methylation, epithelial-mesenchymal transition, tumor microenvironment, progression

## Abstract

TGFβ signaling plays a key role in cancer progression and by shaping tumor architecture and inhibiting the anti-tumor activity of immune cells. It was reported that high expression of TGFβ can promote the invasion and metastasis of cancer cells in a variety of tumors. However, there are few studies on TGFβ2 and its methylation in gastric cancer. We analyzed the Harbin Medical University Cancer Hospital (HMUCH) sequencing data and used public data to explore the potential function and prognostic value of TGFβ2 and its methylation in gastric cancer. In this study, we used the ssGSEA algorithm to quantify 23 methylation sites related to TGFβ2. Survival analysis showed that high expression of TGFβ2 and hypomethylation levels of TGFβ2 were negative factors in the prognosis of gastric cancer. Functional enrichment analysis of methylation revealed that methylation of different TGFβ2 methylation scores was mainly involved in energy metabolism, extracellular matrix formation and cell cycle regulation. In the gastric cancer microenvironment TGFβ2 was associated with high levels of multiple immune cell infiltration and cytokine expression, and high TGFβ2 expression was significantly and positively correlated with stemness markers, stromalscore and EMT. Gene set enrichment analysis also revealed an important role of TGFβ2 in promoting EMT. In addition, we discussed the relationship between TGFβ2 and immunotherapy. The expression of PD-1, PD-L1 and CTLA-4 was elevated in the TGFβ2 high expression group. Also when TGFβ2 was highly expressed, the responsiveness of immune checkpoint blockade (ICB) was significantly enhanced. This indicates that TGFβ2 may become an indicator for predicting the efficacy of immunosuppressive agents and a potential target for immunotherapy.

## Introduction

Gastric cancer is a malignant tumor originating from the epithelium of the stomach, with a high incidence rate and mortality rate (Bray et al., 2018). At present, the conventional treatment of gastric cancer is difficult to remove the tumor cells completely by surgery and chemotherapy, and cancer recurrence often occurs. With the rapid development of medical biotechnology, cancer immunotherapy with strong targeting and low side effects is a rapidly developing research direction in oncology (Cho et al., 2020). Immunotherapy is mainly aimed at immune cells, which can activate the immune system by inhibiting negative immune regulatory factors, enhance the recognition and killing of tumor by immune cells, so as to achieve the purpose of tumor clearance (Cho et al., 2012; Garon, 2017).

In the complex tumor microenvironment, TGFβ is a pleiotropic cytokine involved in the regulation of cancer cell proliferation, apoptosis and metastasis and other cellular processes (David and Massagué, 2018). TGFβ defines three subtypes (TGFβ1, TGFβ2 and TGFβ3), among which TGFβ2 is highly expressed in many cancers, especially those tumors that show high transmission potential (Massagué, 1998). In addition, the increased expression of TGFβ2 in a variety of cancers is often positively correlated with epithelial-mesenchymal transition (EMT) and coordinated with the expression of genes related to driving EMT (Vagenas et al., 2007; Yang et al., 2020). TGFβ signaling in the tumor microenvironment inhibits the anti-tumor function of a variety of immune cell populations, including T cells and natural killer cells, and the resulting immunosuppression severely limits the efficacy of immune checkpoint inhibitors and other immunotherapy approaches. Inhibitors of TGFβ signaling have been evaluated in a number of clinical trials as a major pathway to improve the immune effect of cancer, and combining TGFβ related signals can enhance the effect of other immunosuppressants (Holmgaard et al., 2018; Tauriello et al., 2018). Trabedersen (AP-12009) was an antisense molecule complementary to the mRNA expressed by human TGFβ2 gene, it had been applied II/Phase III clinical cases and has achieved encouraging results (Schlingensiepen et al., 2006).

However, the potential functions and mechanisms of TGFβ2 and TGFβ2 methylation involved in gastric cancer progression are unclear. In this study, we comprehensively analyzed the relationship between TGFβ2 and TGFβ2 methylation levels and the microenvironmental characteristics of gastric cancer based on sequencing data from the Harbin Medical University Cancer Hospital (HMUCH) and public databases. The expression levels of TGFβ2 were also validated in gastric cancer tissues. The results showed that TGFβ2 was highly expressed in gastric cancer and was a poor prognostic factor. TGFβ2 was closely related to the gastric cancer microenvironment, and functional enrichment analysis of TGFβ2 and TGFβ2-associated methylation was performed to explore the possible mechanisms of TGFβ2 action in gastric cancer.

## Materials and Methods

### Patient and Clinical Databases

In this study, frozen tissues (cancer and normal paracancerous tissue more than 5 cm from the tumor cut edge) were collected from 231 gastric cancer surgery patients and subjected to high-throughput sequencing of the transcriptome. All gastric cancer tissues were certified by independent examination by two pathologists to confirm the histological type. Patients were not treated preoperatively with adjuvant therapy such as radiotherapy and chemotherapy. We uploaded and stored the sequencing data into the GEO Datasets (GSE184336). All patients signed an informed consent form. This study complied with the requirements of the Research Ethics Committee of the Harbin Medical University Cancer Hospital (2019-164-R).

We downloaded gene expression data for pan-cancer from the TCGA public database, and the cancer abbreviations and full names for pan-cancer are shown in Supplementary Table S1. The STAD download data also included mutation information, pathology data, and survival status. The GSE84437, GSE63089, GSE62254, GSE34942, GSE29272, GSE26253 and GSE15459 gastric cancer datasets were downloaded from the Gene Expression Omnibus (GEO) database for further analysis (Li et al., 2014; Chia et al., 2015; Cristescu et al., 2015; Zhang et al., 2015; Oh et al., 2018; Subhash et al., 2018; Yoon et al., 2020).

### Western Blot Analysis and Immunohistochemistry

Gastric cancer paraffin blocks were serially sectioned with a section thickness of 4 μm. Immunohistochemical staining was performed as described previously (Kalantari et al., 2022). Total proteins of gastric cancer and normal tissues adjacent to the cancer were extracted and their concentrations were determined. PVDF membranes (Merck Millipore) were blocked with 5% skim milk powder and incubated with TGFβ2 antibody dilution (Proteintech, 19999-1-AP, 1:800) overnight at 4°C environment.

### TGFβ2 Related Methylation

First, we assigned DNA methylation values for TGFβ2 with the average beta value of the probes mapped to the promoter region, including TSS200 (region from -200 bp upstream to the transcription start site (TSS) itself), TSS1500 (from -200 to -1,500 bp upstream of TSS), 1stExon (the first exon) and 5′UTR. Genome annotation of the CpG sites was based on GRCh38. methylation levels of the CpG sites were estimated as beta values (Ding et al., 2020).

Then we used the 23 methylation sites of TGFβ2 as a joint feature of TGFβ2 methylation and used these 23 methylation sites as a dataset for TGFβ2 methylation scoring. The single sample gene set enrichment analysis (ssGSEA) algorithm in the R package GSVA was used to calculate the TGFβ2 methylation score in each sample (Hänzelmann et al., 2013). Patients with STAD were grouped according to the median TGFβ2 methylation score and subjected to methylation differential analysis, and R package methylGSA was used for functional enrichment analysis of methylation (Geeleher et al., 2013).

### Estimate, EMT and mRNAi

The R package ESTIMATE was used to calculate stromalscore, immunescore and ESTIMATEScore to assess the tumor microenvironment, where tumor purity = cos (0.6049872018 + 0.0001467884 * estimate score) (Yoshihara et al., 2013). We also used the ssGSEA algorithm to assess the EMT score in gastric cancer patients with the EMT gene set as described previously (Cristescu et al., 2015). We used the mRNAsi index to assess the tissue stemness characteristics of gastric cancer, and miRNA values were referenced to previous reports (Thorsson et al., 2018).

### Immune Cell Infiltration and Cytokines

We quantified the level of immune cell infiltration in STAD using multiple methods, including CIBERSORT, MCPcounter, TIMER, ssGSEA and quanTIseq algorithms, and immune cells for each STAD patient were calculated as described previously (Becht et al., 2016; Charoentong et al., 2017; Li et al., 2017; Chen et al., 2018; Finotello et al., 2019). In addition, we analyzed the relationship between TGFβ2 and cytokines (receptor, chemokine, immunoinhibitor, immunostimulator and MHC) in the gastric cancer microenvironment (Ru et al., 2019).

### Gene Set Enrichment Analysis

GSEA was presented between the high and low TGFβ2 groups. Pathways with nominal *p* < 0.05 and false discovery rate (FDR) < 0.05 were considered signifificantly enriched (Subramanian et al., 2005). The “h.all.v7.1. entrez.gmt” was chosen as the reference.

### Tumor Mutations

The R package maftools was used to analyze gene mutation information in STAD patients (Mayakonda et al., 2018). The Gistic 2.0 algorithm was used to identify copy number variation (CNV) and to display the frequency of CNV changes between different TGFβ2 groupings (Mermel et al., 2011). Tumor mutation burden (TMB) and neoantigens were calculated as previously described. The SCNA module in the TIMER database (http://timer.cistrome.org/) was used to compare the relationship between different somatic copy number alterations of TGFβ2 and immune cell infiltration.

### Immunotherapy and Chemotherapy

In this study, we evaluated the relationship between TGFβ2 expression levels and immunotherapy. First, we used the submap algorithm to compare the similarity between the STAD expression data and the skin cancer immunotherapy dataset, a feature that can be reflected in the response of STAD to immunotherapy (Hoshida et al., 2007; Roh et al., 2017). We also used the ImmuCellAI database to predict immune checkpoint blockade (ICB) response (anti-PD-1 or anti-CTLA-4 treatment) in STAD patients (Miao et al., 2020). We estimated the sensitivity of STAD patients to chemotherapeutic agents using the genomics of drug sensitivity in cancer (GDSC) database (Yang et al., 2013). The half-maximal inhibitory concentration (IC50) was quantified and analyzed by the R package RpRophetic (Geeleher et al., 2014).

### Nomogram and Calibration

Independent risk factors were identified by cox multi-factor regression analysis, and used R package rms to construct a nomogram to predict the probability of overall survival (Shariat et al., 2008). The calibration chart was used to evaluate the performance of the nomogram, and the 45° diagonal line represented the best predicted value. The index of concordance (C-index) was used to assess the agreement between the actual results and the probabilities predicted by the model.

### Statistical Analysis

The chi-square test was used to analyze the association between different TGFβ2 subgroups and clinicopathological parameters. Kaplan-Meier survival curves were used to compare survival analyses between different subgroups followed by a log-rank test. The R package DESeq2 was used for differential analysis of count data of STAD patients, and the limma package was used for differential analysis of methylation between different subgroups. The area under curve (AUC) value of ROC curve was calculated by the survivalROC R package. All statistical analyses were performed in R software (version 3.6.1). *p*-values less than 0.05 were considered statistically significant differences.

## Result

### TGFβ2 and TGFβ2 Methylation

**FIGURE 1:**
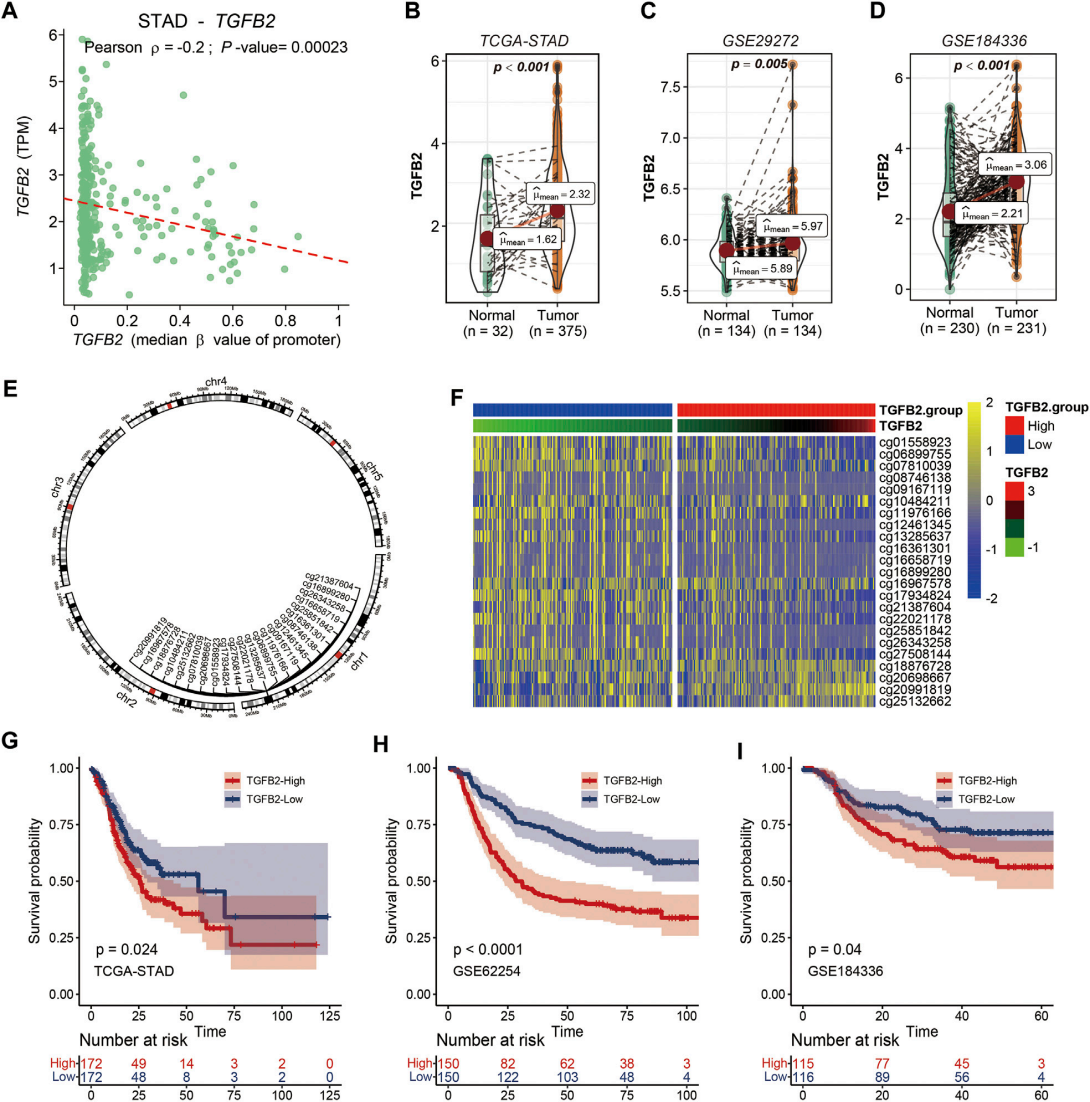
TGFβ2 and TGFβ2 Methylation. **(A)** Relationship between TGFβ2 and TGFβ2 methylation promoter region. **(B–D)** TGFβ2 expression in gastric cancer tissues and normal tissues in STAD, GSE29272 and GSE184336 (HMUCH). **(E)** Location of the TGFβ2 methylation site on the chromosome. **(F)** Heatmap of TGFβ2 and TGFβ2 methylation expression. Kaplan-Meier survival analysis of TGFβ2 in STAD **(G)**, GSE62254 **(H)** and GSE184336 **(I)**.

**FIGURE 2:**
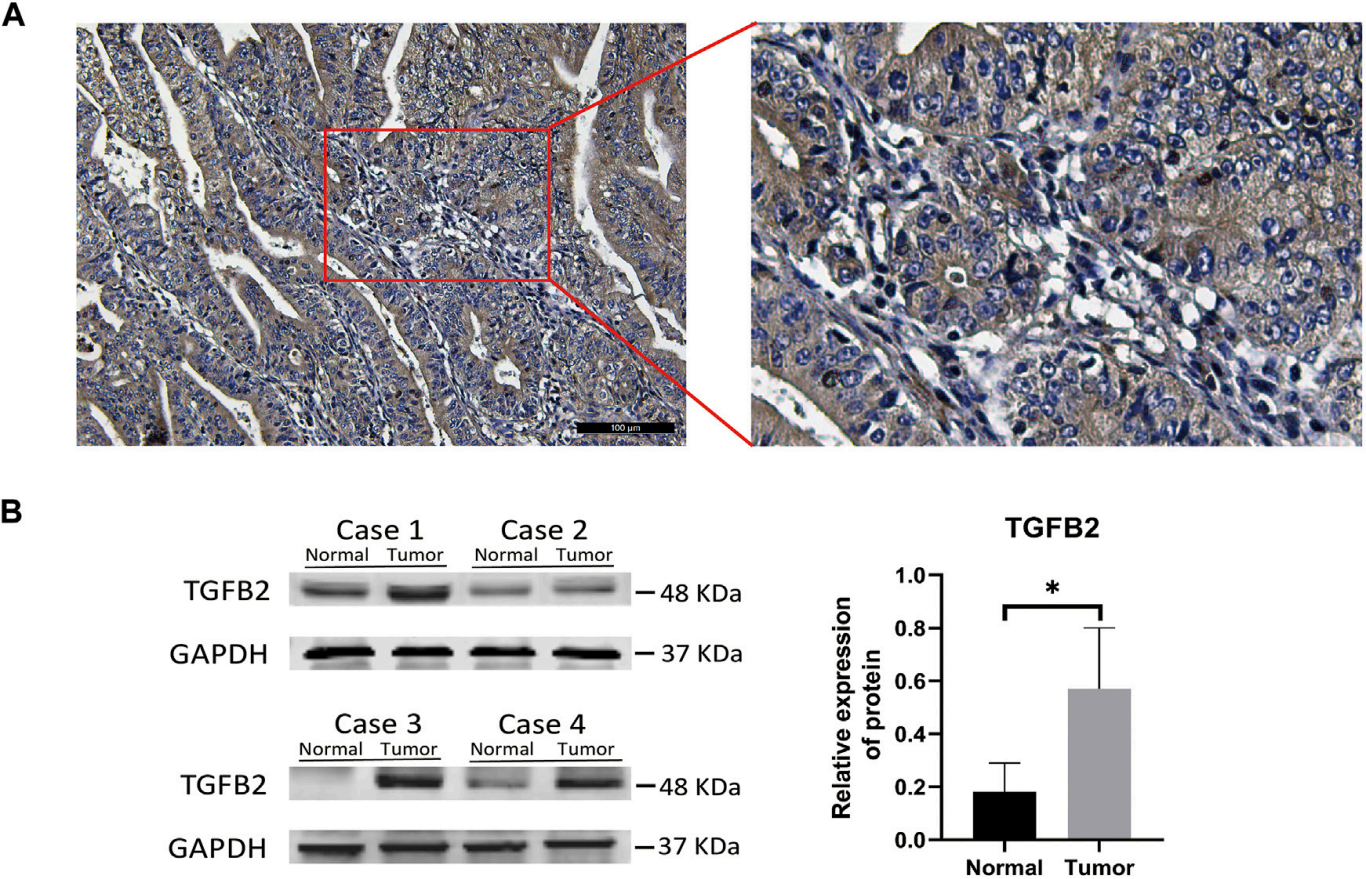
TGFβ2 protein level detection in gastric cancer. IHC **(A)** and Western blot validation **(B)** of TGFβ2 protein in gastric cancer tissues. *p < 0.05.

The correlation between TGFβ2 and promoter region methylation in STAD was significantly negative, while TGFβ1 and TGFβ3 gene expression was not associated with promoter region methylation (Figure 1A and Supplementary Figures S1A,B). We compared the expression level of TGFβ2 in the STAD, GSE29272 and GSE184336 (HMUCH) data sets, and TGFβ2 was expressed at higher levels in cancer tissues than in the adjacent normal tissues (*p* < 0.01) (Figures 1B–D). We also showed the location distribution and detailed information of 23 TGFβ2 methylation sites in the chromosome (Figure 1E and Supplementary Table S2). Heatmap demonstrated TGFβ2 expression and 23 methylation site levels (Figure 1F). In the survival analysis of the STAD, GSE62254 and GSE184336 datasets, patients with high TGFβ2 expression all had shorter survival times (*p* < 0.001) (Figures 1G–I). We verified the protein expression level of TGFβ2 in gastric cancer tissues, and the results of IHC experiments showed that TGFβ2 was mainly distributed in cancer cells, with a small amount of distribution in the mesenchyme (Figure 2A). The results of Western blot assay showed that the protein expression level of TGFβ2 was higher in gastric cancer tissues than normal tissues, which was consistent with the results of transcriptome sequencing level (GSE184336) (Figure 2B).

**FIGURE 3:**
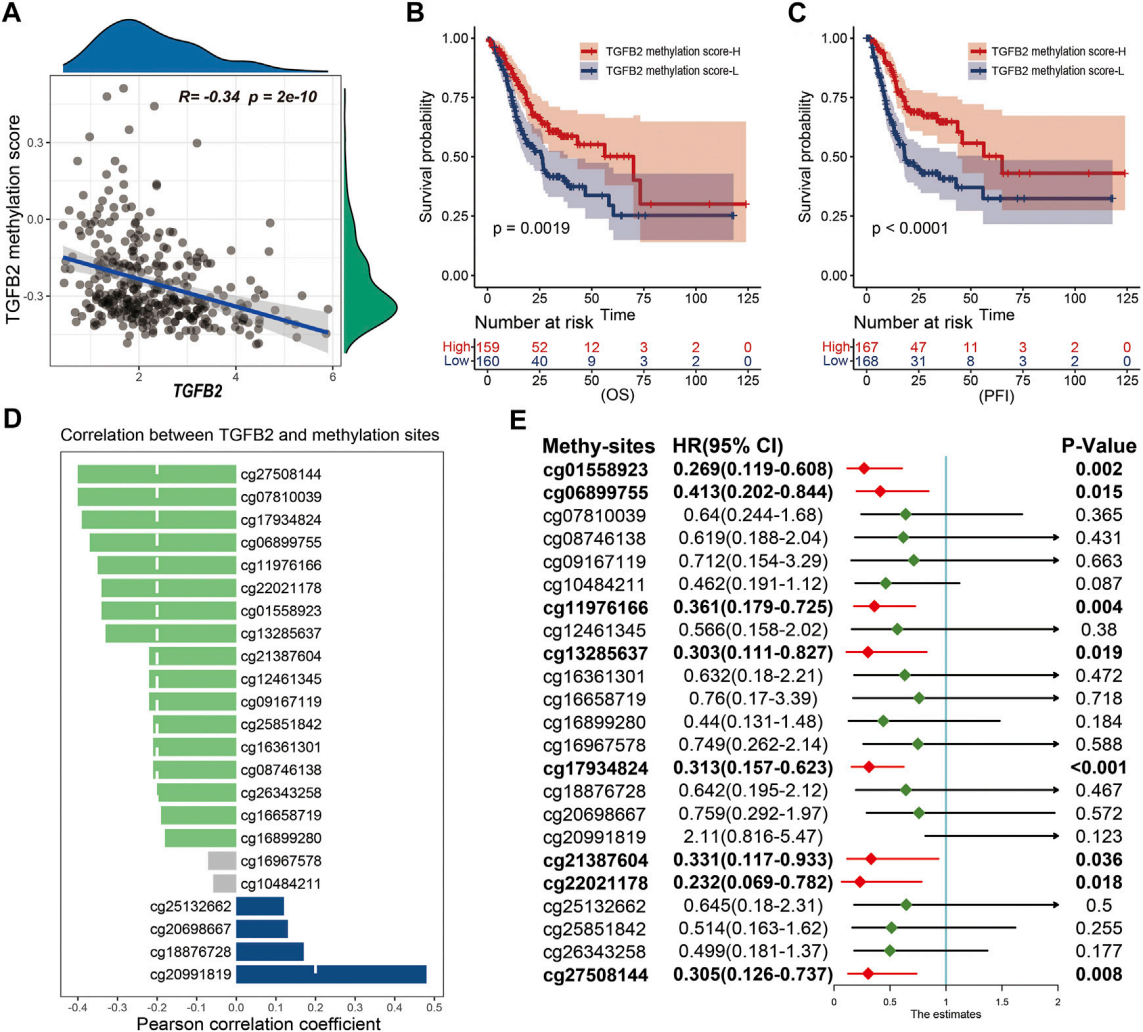
TGFβ2 methylation score. **(A)** Correlation between TGFβ2 methylation score and TGFβ2 expression. Kaplan-Meier survival analysis of TGFβ2 methylation score in OS **(B)** and PFI **(C)**. **(D)** Correlation of TGFβ2 with methylation sites, and the column color represents the correlation of the difference, no statistical significance in gray. **(E)** Prognostic analysis of TGFβ2 methylation sites. HR < 1.0 (Red) indicates that the methylation site is a favorable prognostic marker.

We performed cox univariate analysis of the methylation sites and the results showed that cg01558923, cg06899755, cg11976166, cg13285637, cg17934824, cg21387604, cg22021178 and cg27508144 were statistically significant in STAD (Figure 3E). In addition, we also performed Kaplan-Meier survival analysis for 23 methylation sites, and most of them (cg01558923, cg06899755, cg08746138, cg10484211, cg11976166, cg12461345, cg13285637, cg17934824, cg21387604, cg22021178, cg26343258 and cg27508144) high expression was beneficial to prolong patient survival (Supplementary Figure S2).

### TGFβ2 Methylation Score

To quantify the overall expression level of TGFβ2 methylation, we evaluated TGFβ2 methylation expression in STAD patients using the ssGSEA algorithm with 23 methylation sites as the reference set. Pearson correlation analysis showed a significant negative correlation between TGFβ2 and TGFβ2 methylation score (Figure 3A). Patients with high TGFβ2 methylation scores in STAD had longer survival times at OS (overall survival), PFI (progression-free interval), DPI (disease-free interval) and DSS (disease-specific survival) levels (Figures 3B,C and Supplementary Figures S1C,D). In addition, we also analyzed the correlation between 23 TGFβ2 methylation sites and TGFβ2 expression separately, and the results showed that 18 methylation sites were expressed at opposite levels to TGFβ2 expression. Although the levels of the four methylation sites (cg18876728, cg20698667, cg25132662 and cg20991819) followed the same trend as TGFβ2 expression, the cg18876728, cg20698667 and cg25132662 site was in the body region and the cg20991819 was in the 3′-UTR region at the end of the coding region, neither of which was within the promoter region that affects TGFβ2 expression (Figure 3D and Supplementary Figure S3).

### Differential Analysis of Methylation and Functional Enrichment Analysis

**FIGURE 4:**
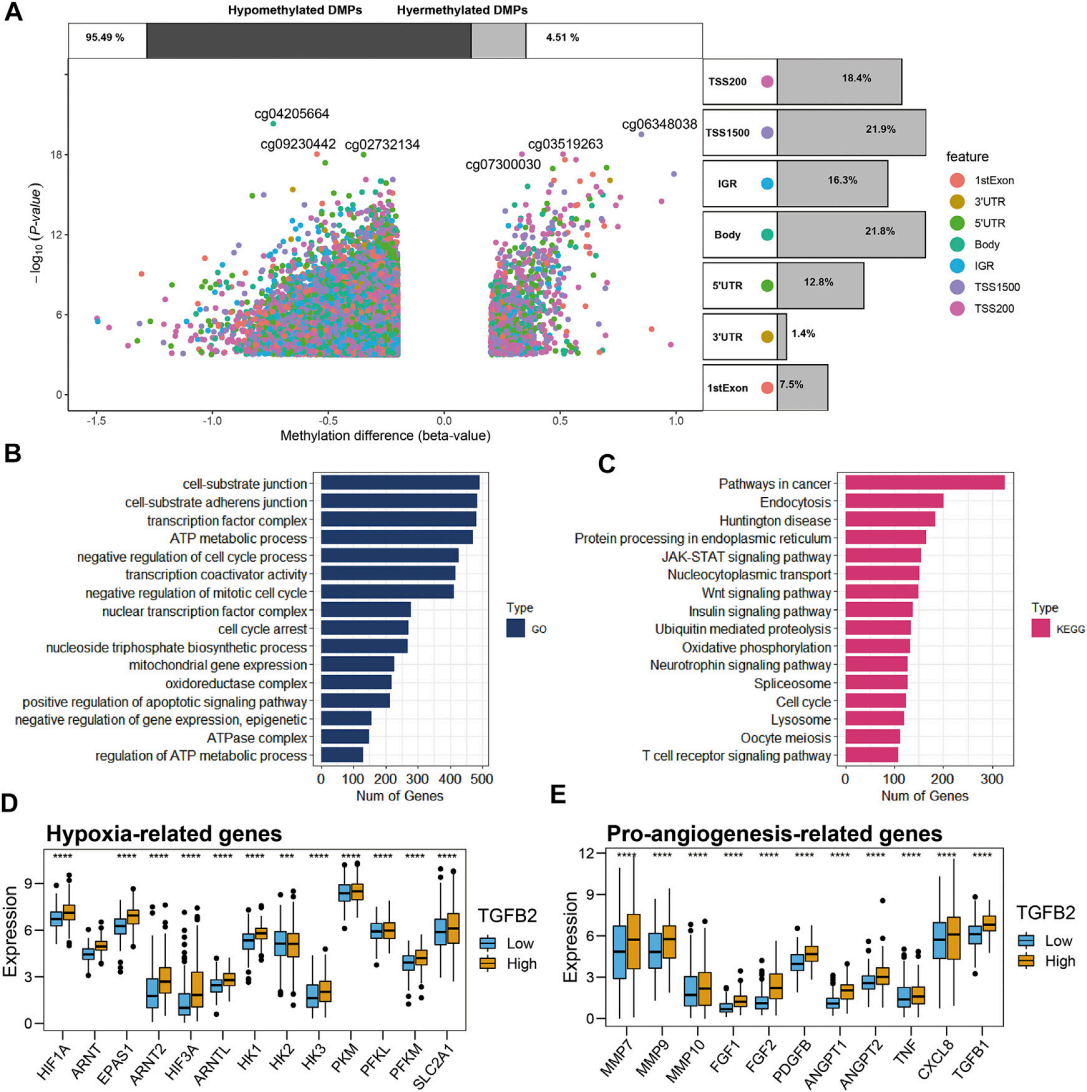
Differential analysis of methylation and functional enrichment analysis. **(A)** Methylation volcano plots for different TGFβ2 methylation score groupings, with the location information of the methylation sites annotated on the right side of the figure notes and the expression differences of methylation on the top. **(B,C)** Methylation functional enrichment analysis, GO and KEGG results. Comparison of hypoxia-related genes **(D)** and pro-angiogenesis-related genes **(E)** in different TGFβ2 subgroups. *p < 0.05, **p < 0.01, ***p < 0.001, and ****p < 0.0001.

We grouped STAD patients according to the median TGFβ2 methylation score, 169 cases in the TGFβ2-methy.score-Low group and 168 cases in the TGFβ2-methy.score-High group. After removing some undetected methylation probes, a total of 337 samples (pathological type of gastric adenocarcinoma) and 375,361 DNA methylation sites were obtained from TCGA. We identified 25,053 differentially methylated positions (DMPs) in the analysis of differences between different TGFβ2 methylation score subgroups with screening criteria of *p* < 0.05 and |delta| > 0.2 (Figure 4A).

Then, we performed functional enrichment analysis of methylation among different TGFβ2 methylation score groupings based on differential methylation analysis. The results of GO (Gene Onotology) analysis showed that methylation between different subgroups is mainly involved in cell cycle regulation (cell cycle arrest, nuclear transcription factor complex and negative regulation of mitotic cell cycle), extracellular matrix formation (cell-substrate adherens junction and cell-substrate junction) and energy metabolism regulation (regulation of ATP metabolic process, ATPase complex, oxidoreductase complex, mitochondrial gene expression, nucleoside triphosphate biosynthetic process and ATP metabolic process) (Figure 4B). The results of KEGG (Kyoto Encyclopedia of Genes and Genomes) analysis showed that methylation among different subgroups may be involved in cancer progression by regulating signaling pathways Wnt signaling pathway, JAK-STAT signaling pathway and Pathways in cancer (Figure 4C).

### TGFβ2 and Hypoxia and Pro-Angiogenesis Genes

Given the important function of methylation on metabolic regulation among different TGFβ2 methylation score subgroups and that oxygen content is one of the important factors affecting energy metabolism, we compared hypoxia and proangiogenesis-related genes among patients with different TGFβ2 subgroups. The results showed that hypoxia-related genes (HIF1A family, HK1, HK3, PKM, PFKL, PFKM and SLC2A1) were higher in the high TGFβ2-expressing subgroup of patients, while pro-angiogenic genes (MMP7, MMP9, MMP10, FGF1, FGF2, PDGFB, ANGPT, ANGPT2, TNF, CXCL8 and TGFβ1) expression was also higher (Figures 4D,E).

### TGFβ2 and Gastric Cancer Microenvironment

**FIGURE 5:**
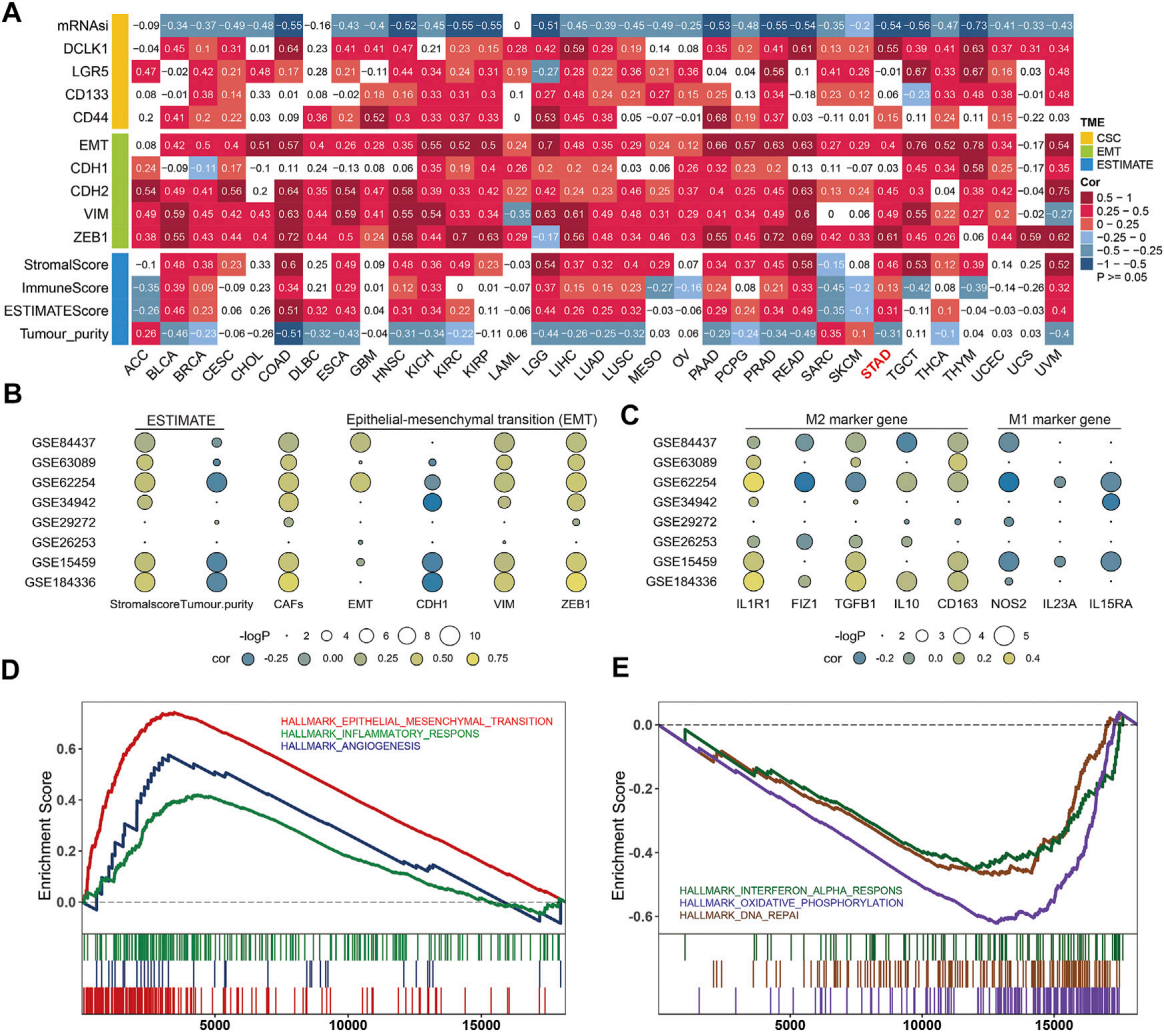
TGFβ2 and gastric cancer microenvironment. **(A)** Correlation analysis of TGFβ2 with CSC, EMT and ESTIMATE in The Cancer Genome Atlas (TCGA). **(B)** Bubble plots of the correlation between TGFβ2 and ESTIMATE (Stromalscore and Tumor purity), CAFs and EMT (EMT, CDH1, VIM, ZEB1) in multiple GEO datasets. **(C)** Bubble plots of TGFβ2 correlation with M1 (IL1R1, FIZ1, TGFβ1, IL10 and CD163) and M2 (NOS2, IL23A and IL15RA) marker genes in multiple GEO datasets. Different colors represent different correlations. **(D,E)** Gene Set Enrichment Analysis (GSEA) was used to analyze the differences in gene function among different TGFβ2 subgroups.

In the tumor microenvironment we analyzed the relationship between TGFβ2 and tumor stemness, EMT and ESTIMATE scores, respectively, with similar results between different pan-cancers. TGFβ2 was significantly negatively correlated with mRNAsi and significantly positively correlated with cancer stem cell markers DCLK1, Lgr5, CD133 and CD44. Also TGFβ2 was significantly positively correlated with EMT score and mesenchymal markers (CDH2, VIM and ZEB1), and in STAD, there was no significance in the analysis between TGFβ2 and CDH1. In addition, TGFβ2 was positively correlated with stromalscore, immunescore and ESTIMATEScore and negatively correlated with tumor purity in a variety of tumors including STAD (Figure 5A).

We also performed validation in the GEO gastric cancer dataset (GSE184336 (HMUCH), GSE84437, GSE63089, GSE62254, GSE34942, GSE29272, GSE26253 and GSE15459). The results showed that TGFβ2 was positively correlated with stromalscore and CAFs and negatively correlated with tumor purity in several datasets, which was consistent with the results of the analysis in STAD. In the correlation analysis with EMT, TGFβ2 was positively correlated with EMT and mesenchymal markers and negatively correlated with CDH1 in multiple datasets (Figure 5B). We also analyzed the relationship between TGFβ2 and M1 and M2 marker genes, and the results showed that TGFβ2 was negatively associated with most M1-related marker genes and positively associated with M2-related genes (Figure 5C). According to the differential analysis of different TGFβ2 groupings, GSEA analysis showed that the high TGFβ2 expression group promoted epithelial-mesenchymal transition, inflammatory response and angiogenesis, and inhibited interferon alpha response, oxidative phosphorylation and DNA repair (Figures 5D,E).

### TGFβ2 and Immune Cell Infiltration and Cytokine Levels

**FIGURE 6:**
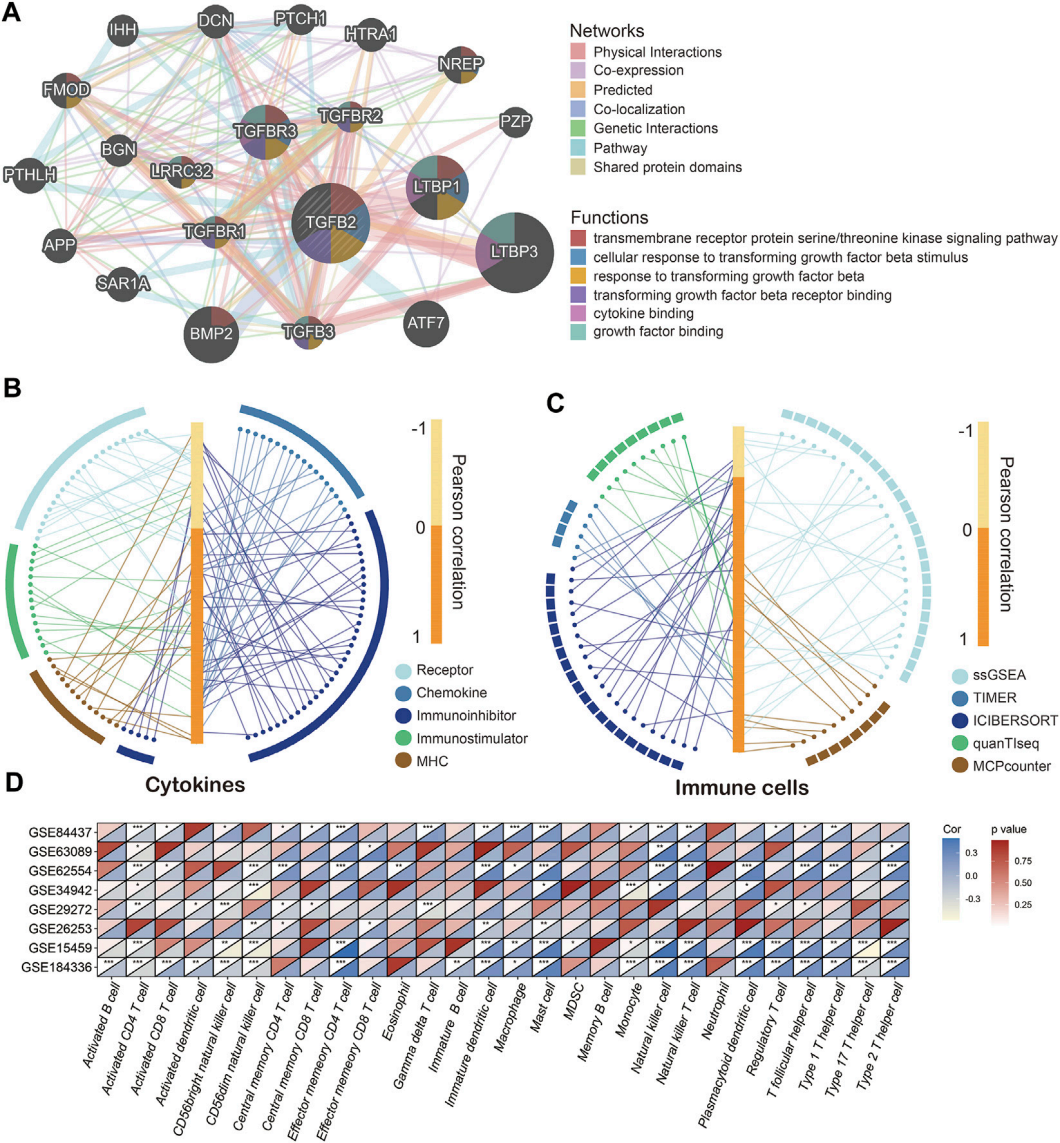
TGFβ2 and immune cell infiltration and cytokine levels. **(A)** TGFβ2-related gene network. Correlation of TGFβ2 with immune cells **(B)** and cytokines **(C)**. Correlation of TGFβ2 with immune cells in multiple GEO datasets **(D)**. *p < 0.05, **p < 0.01, and ***p < 0.001.

We used the GeneMANIA cloud database to analyze other genes related to TGFβ2, and the gene network showed that TGFβ2 was more closely related to LTBP3, LTBP1 and BMP2 (Figure 6A) (Warde-Farley et al., 2010). Given the important role of TGFβ2 in the tumor microenvironment, we further analyzed the relationship between TGFβ2 and immune cell infiltration and cytokines (receptor, chemokine, immunoinhibitor, immunostimulator and MHC). The results showed that TGFβ2 was significantly and positively correlated with increased levels of multiple immune cell infiltration and cytokines (Figures 6B,C). Meanwhile, TGF-β2 had strong correlation with various immune cell (CD8^+^ T cell, Monocyte, TAM, M1 Macrophage, M2 Macrophage and Treg) marker genes (Supplementary Table S3).

Similarly in the high TGFβ2 subgroup most immune cells infiltrated and cytokine expression levels were higher (Supplementary Figures S4A–E and Supplementary Figure S5). In contrast, most immune cells in the high TGFβ2 methylation score subgroup had low levels of infiltration (Supplementary Figure S4F). We also analyzed the correlation between TGFβ2 and immune cell infiltration in the GEO dataset, and the results showed that TGFβ2 positively correlated with activated CD4 T cell, CD56dim natural killer cell, immature dendritic cell, mast cell, natural killer cell and regulatory T cell in multiple GEO datasets (Figure 6D).

### TGFβ2 and Mutations

**FIGURE 7:**
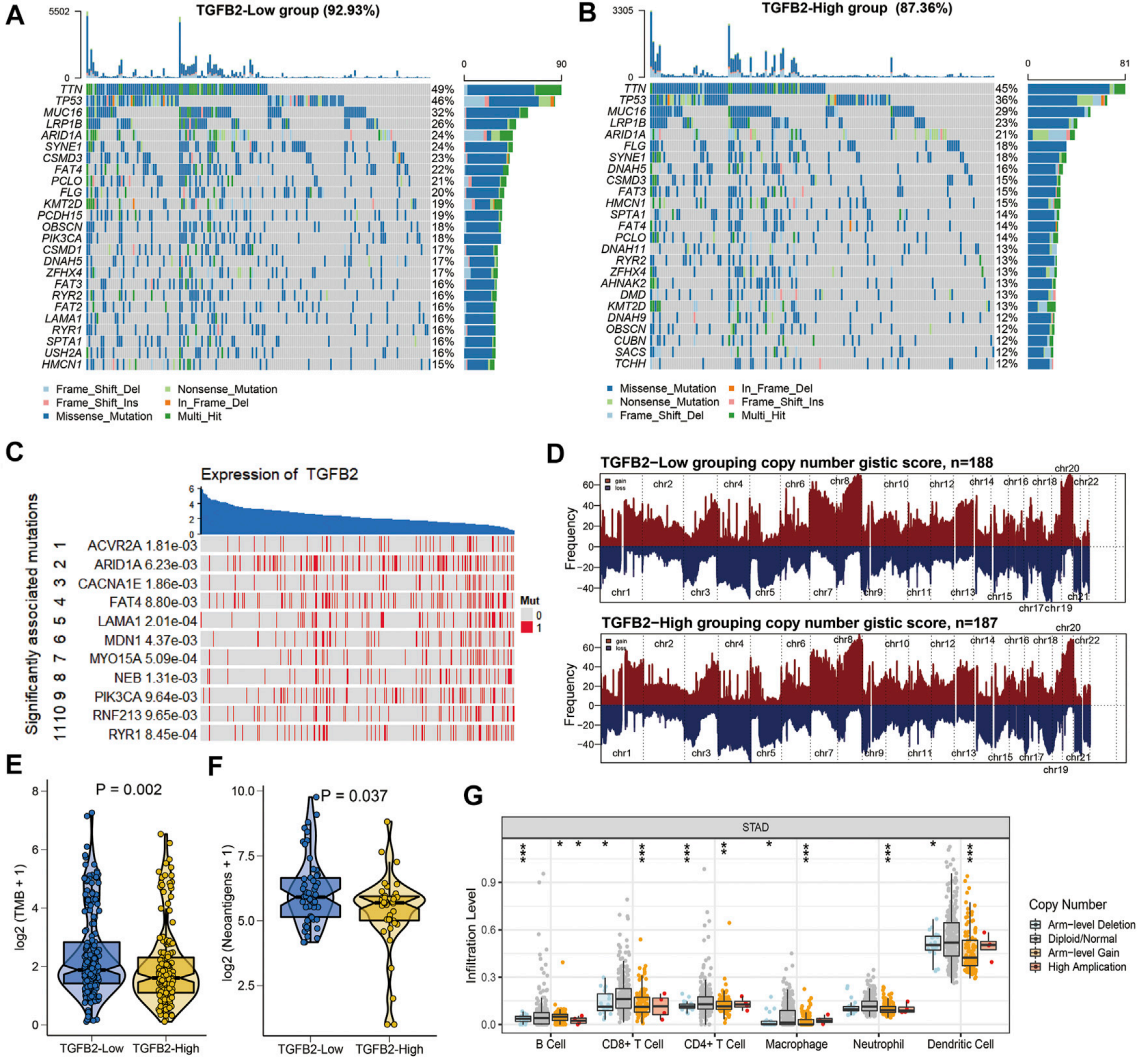
TGFβ2 and mutations. **(A, B)** Waterfall plot of the 25 genes with the highest mutation frequencies in different TGFβ2 groups. **(C)** Mutations in genes affecting TGFβ2 expression. **(D)** Comparison of the frequency of copy number changes in different TGFβ2 groupings. Chromosomal locations of peaks of significantly recurring focal amplification (red) and deletions (blue) were presented. Comparison of TMB **(E)** and neoantigens **(F)** between different TGFβ2 groups. **(G)** Effect of the Genetic Alterations of TGFβ2 on the Immune Cell Infifiltration. *p < 0.05, **p < 0.01, and ***p < 0.001.

We showed the 25 genes with the highest mutation frequency in different TGFβ2 subgroups and the difference of copy number variation between the two groups (Figures 7A,B,D). TGFβ2 expression was associated with mutations in genes (ACVR2A, ARID1A and CACNA1E) (Figure 7C). Tumor mutation burden and neoantigens were both higher in the TGFβ2 low expression group (Figures 7E,F). We also analyzed the effect of somatic copy number alterations (CNAs) of TGFβ2 on immune cell infiltration to elucidate the potential mechanism of TGFβ2 associated with infiltration of different immune cells, and the results showed that arm-level deletion and arm-level gain significantly affected the infiltration levels of B cells, CD4^+^ T cells, CD8^+^ T cells, neutrophils, macrophages and dendritic cells in STAD (Figure 7G).

### TGFβ2 and Immunotherapy and Chemotherapy

**FIGURE 8:**
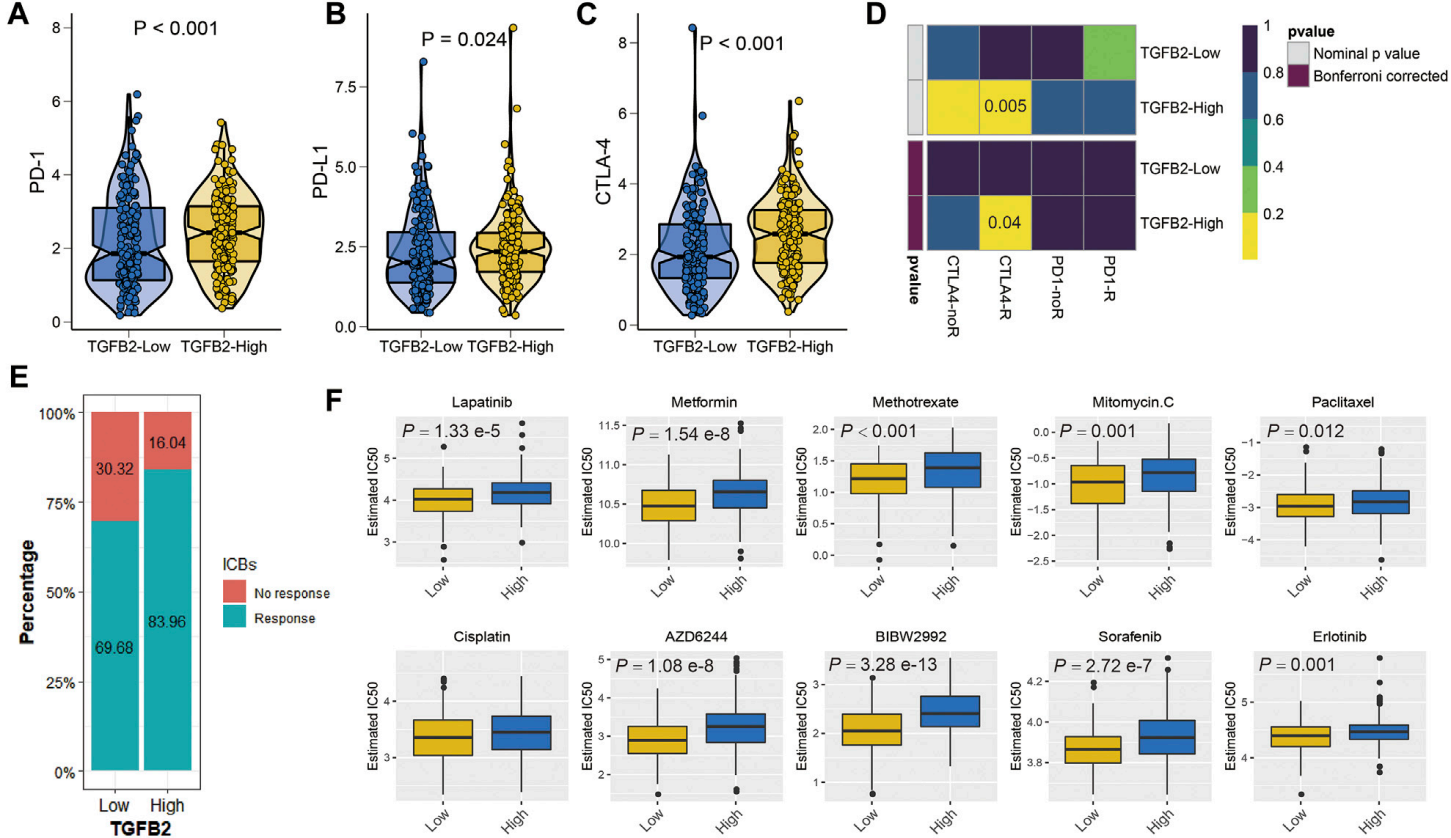
TGFβ2 and immunotherapy and chemotherapy. Differences in immune checkpoints between high and low TGFβ2 groups, PD-1 **(A)**, PD-L1 **(B)** and CTLA-4 **(C)**. **(D)** Heatmap visualized the response to anti-CTLA4 and anti-PD1 therapies between the two groups. **(E)** The histogram showed the responsiveness of immunotherapy between high and low TGFβ2 groups, the height of each bar represents the frequency of change. **(F)** Boxplots depicted the differences in the estimated IC50 levels of Lapatinib, Metformin, Methotrexate, Mitomycin. C, Paclitaxel, Cisplatin, AZD6244, BIBW2992, Sorafenib and Erlotinib between the high and low TGFβ2 groups.

We explored the relationship between TGFβ2 and immunotherapy, and PD-1, PD-L1 and CTLA-4 expression levels were higher in the TGFβ2 high expression group (*p* < 0.05) (Figures 8A–C). We evaluated immunotherapy response using two methods, firstly the submap algorithm results showed that CTLA-4 immunosuppressive treatment was meaningful for patients in the high TGFβ2 group and secondly a higher proportion of patients in the high TGFβ2 group were also predicted to respond to immunotherapy according to the ImmuCellAI database (chi-squared test = 10.724, *p* = 0.001) (Figures 8D,E).

As an important method of adjuvant treatment for gastric cancer, chemotherapy plays an important role in clinical treatment. Differences in chemotherapy (IC50) between different TGFβ2-expressing groups were predicted according to the GDSC database, which showed a higher sensitivity to Lapatinib, Metformin, Methotrexate, Mitomycin. C, Paclitaxel, AZD6244, BIBW2992, Sorafenib and Erlotinib in the low TGFβ2 group (Figure 8F).

### TGFβ2 and TGFβ2 Methylation Scores and Clinicopathological Factors

**FIGURE 9:**
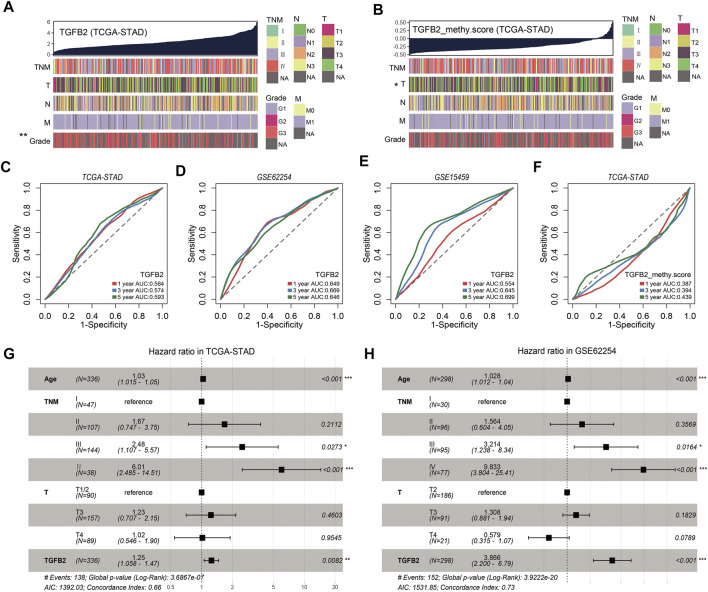
TGFβ2 and TGFβ2 methylation scores and clinicopathological factors. **(A)** Heatmap of TGFβ2 and clinicopathological features in STAD. **(B)** Heatmap of TGFβ2 methylation score and clinicopathological features in STAD. The predictive value of TGFβ2 in patients in cohorts STAD **(C)**, GSE62254 **(D)** and GSE15459 **(E)**. The predictive value of TGFβ2 methylation scores in patients in cohorts STAD **(F)**. Multivariate Cox regression model analysis, which included the factors of age, TNM stage, T stage and TGFβ2 in the STAD **(G)** and GSE62254 **(H)** cohorts. *p < 0.05, **p < 0.01, and ***p < 0.001.

To further assess the clinical relevance of TGFβ2 and TGFβ2 methylation scores, we divided patients into two groups based on median TGFβ2 and TGFβ2 methylation scores (Table 1 and Supplementary Table S4 **)**. In the STAD dataset, patients in the TGFβ2 high expression group had worse tumor grade, and the TGFβ2 methylation score was associated with T stage (Figures 9A,B). The time-dependent area under the ROC curve (AUC) for TGFβ2 at 1, 3 and 5 years in the STAD, GSE62254 and GSE15459 datasets was around 0.6 (Figures 9C–E). In addition, The AUCs of the 1-, 3- and 5-years time-dependent ROC curves for TGFβ2 methylation scores in STAD were 0.387, 0.394 and 0.439, respectively (Figure 9F).

**TABLE 1:** The relationship between TGFβ2 expression and clinicopathological factors in HMUCH (GSE184336) and TCGA-STAD database.

Clinical Features	Total	TGFβ2 Expression (HMUCH)		*p*-Value	Total	TGFβ2 Expression (STAD)		*p*-Value
		Low (%)	High (%)			Low (%)	High (%)	
Age				0.948				0.864
<60	114	57 (49.1)	57 (49.6)		105	56 (29.8)	56 (30.6)	
≥60	117	59 (50.9)	58 (50.4)		236	132 (70.2)	127 (69.4)	
Gender				0.462				0.859
Female	83	39 (33.6%)	44 (38.3%)		122	68 (36.2)	66 (35.3)	
Male	148	77 (66.4%)	71 (61.7%)		222	120 (63.8)	121 (64.7)	
TNM stage				0.708				0.453
Ⅰ	36	20 (17.2)	16 (13.9)		53	32 (17.5)	21 (11.9)	
Ⅱ	49	27 (23.3)	22 (19.1)		112	58 (31.7)	54 (30.7)	
Ⅲ	129	61 (52.6)	68 (59.1)		154	74 (40.4)	80 (45.5)	
Ⅳ	17	8 (6.9)	9 (7.8)		40	19 (10.4)	21 (11.9)	
T stage				0.217				0.176
T1	21	11 (9.5)	10 (8.7)		19	14 (7.4)	5 (2.8)	
T2	25	17 (14.7)	8 (7)		80	41 (21.8)	39 (21.8)	
T3	142	70 (60.3)	72 (62.6)		168	87 (46.3)	81 (45.2)	
T4	43	18 (15.5)	25 (21.7)		100	46 (24.5)	54 (30.2)	
N stage				0.241				0.494
N0	65	33 (28.4)	32 (27.8)		111	63 (34.6)	48 (27.3)	
N1	29	19 (16.4)	10 (8.7)		97	46 (25.3)	51 (29.0)	
N2	44	23 (19.8)	21 (18.3)		76	36 (19.8)	40 (22.7)	
N3	93	41 (35.3)	52 (45.2)		74	37 (20.3)	37 (21)	
Histologic Grade				0.606				**0.003**
G1	4	3 (2.6)	1 (0.9)		10	7 (3.8)	3 (1.7)	
G2	92	46 (39.6)	46 (40)		138	84 (45.2)	54 (29.8)	
G3	135	67 (57.8)	68 (59.1)		219	95 (51.0)	124 (68.5)	

Bold values indicate *p*-value < 0.05.

To test whether TGFβ2 could be an independent prognostic factor, we performed multivariate Cox regression analysis based on the clinical characteristics of the patients, including age, T stage and TNM stage. We found that TGFβ2 was a reliable and independent prognostic marker for assessing patient prognosis in STAD (HR = 1.25, 95% CI 1.058-1.47, *p* = 0.009; Figure 9G). TGFβ2 remained an independent prognostic marker in the GSE62254 dataset (HR = 3.866, 95% CI 2.2-6.79, *p* < 0.001; Figure 9H). These results suggested that TGFβ2 was a valid predictor of prognosis for patients with gastric cancer.

### Nomogram and Calibration

**FIGURE 10:**
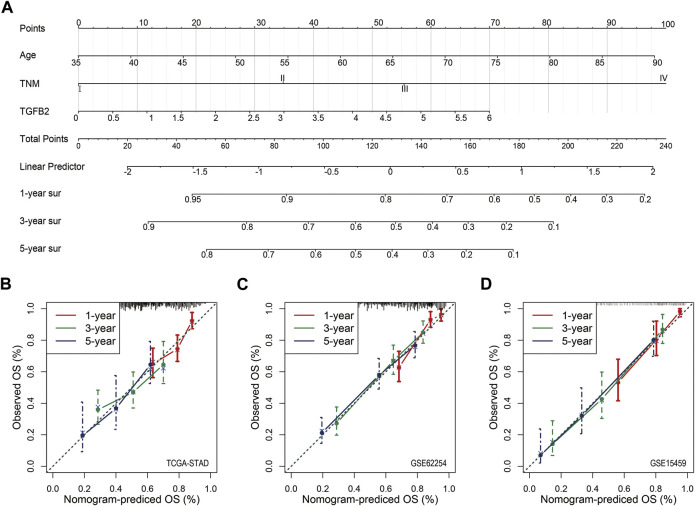
Nomogram and Calibration. **(A)** Nomogram for predicting the probability of 1-, 3- and 5-years OS for STAD patients. Calibration curve for the nomogram for predicting the 1-, 3-, and 5-years OS probabilities for STAD **(B)**, GSE62254 **(C)** and GSE15459 **(D)**.

In order to quantify the influence of clinicopathological factors including TGFβ2 on the prognosis, we used a nomogram to establish a predictive model. We drew a nomogram based on the multivariate analysis of STAD patients (age, TNM and TGFβ2; *p* < 0.05) (Figure 10A). The calibration chart for the 5-years survival rate of the three cohorts was well predicted (C-index: 0.661 for STAD cohort, 0.726 for validation cohort GSE62254, and 0.762 for validation cohort GSE15459) (Figures 10B–D).

## Discussion

Cancer cells are controlled by multiple regulatory signals during development, among which the TGFβ family plays an important role. Some studies have reported that the TGFβ2 signaling pathway plays a pro-cancer role in a variety of tumors, such as highly aggressive gliomas, breast cancers, and squamous cell carcinomas (Busch et al., 2015; Zhang et al., 2017; Abraham et al., 2018). However, there are fewer studies on TGFβ2 and its methylation in gastric cancer. The results of this study showed that the TGFβ2 expression level was significantly and negatively correlated with the TGFβ2 methylation promoter region and TGFβ2 methylation score. The expression levels of TGFβ2 in gastric cancer tissues were significantly higher in the datasets GSE184336, GSE29272 and STAD than in normal tissues adjacent to the cancer. Survival analysis showed that patients with high levels of TGFβ2 gastric cancer had shorter survival times and those with high TGFβ2 methylation scores had longer survival times. Multiple methylation sites acted as conservation roles in the univariate analysis of TGFβ2 methylation sites. TGFβ2 was an independent prognostic factor for patients with gastric cancer in both the STAD and GSE62254 datasets in a multifactorial survival analysis. In view of the relationship between TGFβ2 expression and TGFβ2 methylation, the results all emphasized that high TGFβ2 expression is a poor prognostic factor for gastric cancer, which is consistent with the results of previous research reports (Yang et al., 2020).

Methylation functional enrichment analysis revealed that methylation of different TGFβ2 methylation scores is mainly involved in participating in cell cycle regulation, extracellular matrix formation and energy metabolism regulation. The proportion of energy metabolism-related regulation was high in the enrichment analysis (GO) results, and considering the importance of oxygen content in energy supply, we further analyzed the marker genes that correlate TGFβ2 with the oxygen content of the tissue microenvironment. The results showed high expression of HIF1A family and pro-angiogenesis related genes in the microenvironment of patients in the high TGFβ2 group, which can be inferred to be a hypoxic environment within the high TGFβ2 expressing tumor tissues. Studies have reported that in the myocardial ischemia experiment of mice, with the increase of hypobaric hypoxia time, the mRNA level of TGFβ increases (Xiao et al., 2016). Hypoxia, as one of the important features of tumors, plays an important role in cancer progression; therefore, high expression of TGFβ2 may also be indirectly involved in tumor progression by regulating hypoxia-related genes. Tumor cells induce hypoxia through various mechanisms, such as high metabolic rate and high oxygen consumption, creating a chronic hypoxic environment, activating hypoxia-inducible factors (HIF) signaling pathways, accelerating tumor growth, increasing tumor aggressiveness, and contributing to tumor metastasis (Andrysik et al., 2021).

In the gastric cancer microenvironment TGFβ2 is closely related to tumor stemness, EMT and stroma. Cancer stem cells (CSCs) a subpopulation of tumor cells with the ability to self-renew and differentiate, play an important role in cancer progression (Yang et al., 2015). mRNAsi is an indicator describing the degree of similarity of tumor cells to stem cells and can be considered as a quantification of CSCs. TGFβ2 was negatively correlated with mRNAsi and significantly positively correlated with stemness markers (DCLK1 and CD44) in this study, so TGFβ2 may be important factor in maintaining tumor stemness and promoting tumor differentiation in gastric cancer (Kalantari et al., 2017). EMT is the process by which polar epithelial cells convert to migratory mesenchymal cells and acquire the ability to invade and migrate, and it is present in several physiological and pathological processes in the human body. There are many regulatory factors of EMT, such as TGFβ, Wnt signaling pathway, microRNA and transcription factors (Vergara et al., 2019). Our results showed that TGFβ2 was significantly and positively correlated with EMT, CDH2, VIM and ZEB1. As cancer cells weaken their epithelial features during EMT, they may express fewer tumor-specific neoantigens to avoid recognition by immune cells, all of which contribute to cancer progression (Batlle and Massagué, 2019). Also high expression of TGFβ2 in the gastric cancer microenvironment was associated with higher levels of stromalscore and CAFs infiltration, and similar results were seen in the pan-cancer and multiple gastric cancer datasets. In functional enrichment analysis, the high TGFβ2 expression group promoted epithelial-mesenchymal transition and angiogenesis and inhibited oxidative phosphorylation and DNA repair.

Our findings suggested that TGFβ2 was strongly correlated with a variety of immune cell infiltrates, including CD8^+^ T cells, monocytes, TAM, M1 macrophages, M2 macrophages and Treg. Treg is a subgroup of cells with significant immunosuppressive effects (Flemming, 2016). As an important secretion factor, TGFβ2 has a strong correlation with Treg, so Treg may inhibit protective immune cells through TGFβ2. TGFβ also affects the types of Myeloid-derived suppressor cells (MDSCs) in TME, including macrophages and neutrophils, prompting them to gradually transform into a tumor-promoting phenotype during cancer progression (Fridlender et al., 2009; Laoui et al., 2011). The results of this study showed that TGFβ2 was negatively correlated with M1 macrophage markers and positively correlated with M2 macrophage markers, showing an overall M2 macrophage phenotype that promotes immune escape of tumor cells. The relationship between TGFβ2 and immune cell infiltration was also demonstrated by genetic mutations, and the results showed that the somatic copy number alteration (Arm-level Gain) of the TGFβ2 gene was closely related to the level of STAD immune cell infiltration.

Given the strong correlation between TGFβ2 and immune cell infiltration and its important role in the regulation of immune cell function in gastric cancer, TGFβ2 is an important factor that cannot be ignored in gastric cancer immunotherapy. Stromal fibroblasts and other cells in tumor tissue shape the immunosuppressive environment of the tumor through TGFβ signaling, inhibiting the anti-tumor activity of immune cells and preventing or diminishing the effects of anti-cancer immunotherapy (Chakravarthy et al., 2018). Therefore, inhibition of TGFβ signaling is considered to be a prerequisite and an important way to improve the effectiveness of immunotherapy. Considering TGFβ, CTLA4 and PD-L1/PD-1 as parallel immunosuppressive pathways, combining TGFβ inhibitors with other immune checkpoint inhibitors may improve treatment outcomes. Combination therapy has been pre-evaluated in mouse cancer models where, depending on the model and experimental design, anti-PD-1 or anti-PD-L1 antibody treatment enhanced the antitumor effects of TGFβ inhibition and inhibited tumor metastasis (Mariathasan et al., 2018). The results of this study showed higher TMB and neoantigens in the low TGFβ2 expression group. At the same time, we also predicted the response of TGFβ2 expression level to ICB treatment, and patients with high TGFβ2 expression had a higher response rate from ICB therapy. However, this study has some limitations and still needs further validation by biological experiments and clinical data.

In conclusion, high expression of TGFβ2 and hypomethylation of TGFβ2 are factors of poor prognosis in gastric cancer. The high expression of TGFβ2 in gastric cancer tissue affects the tumor microenvironment and the level of immune cell infiltration by regulating DNA damage, angiogenesis, inflammation and EMT. The high responsiveness of ICB when TGFβ2 is highly expressed suggests that the detection of TGFβ2 expression can predict the response of gastric cancer patients to immune checkpoint inhibitors and may serve as a candidate target for gastric cancer immunotherapy.

## Data Availability

The datasets presented in this study can be found in online repositories. The names of the repository/repositories and accession number(s) can be found in the article/Supplementary Material.
