# Supplementary figures and images for: TGFβ2 is a Prognostic Biomarker for Gastric Cancer and is Associated With Methylation and Immunotherapy Responses
Source: Front Genet. 2022 May 10;13:808041. doi: 10.3389/fgene.2022.808041 (PMC9127534; doi:10.3389/fgene.2022.808041)

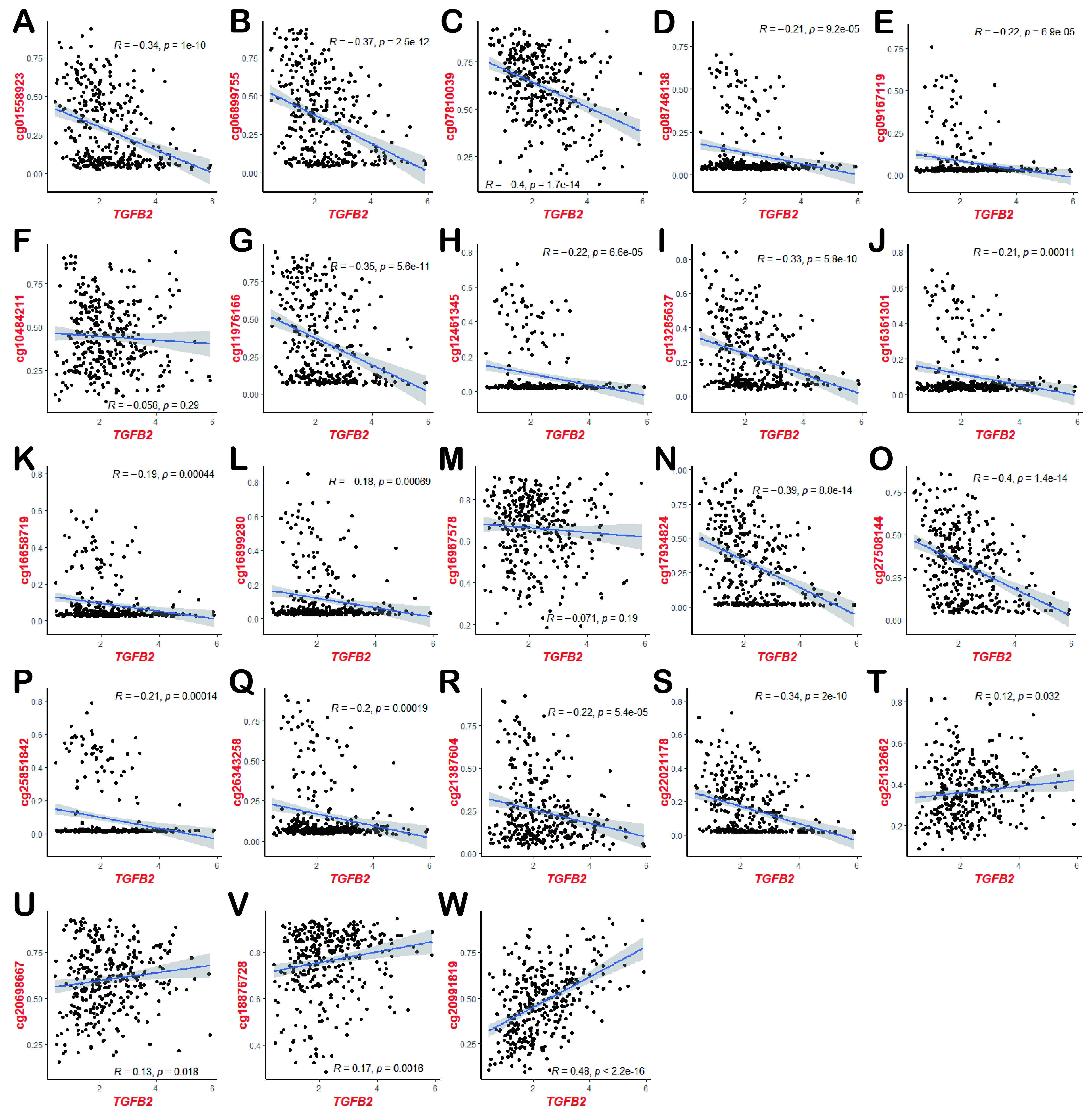

Supplement: Supplementary file 1 [file Image3.JPEG]

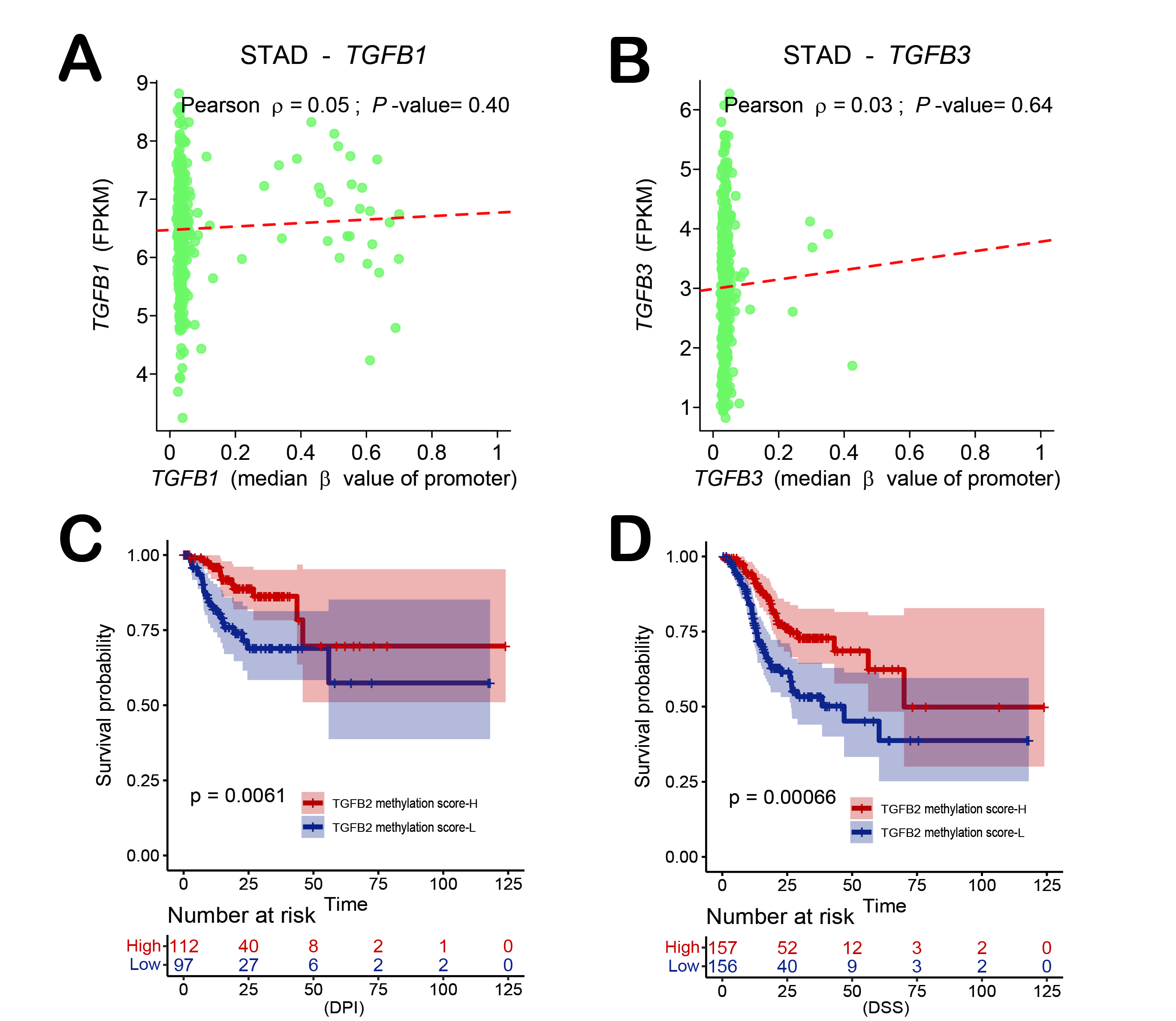

Supplement: Supplementary file 3 [file Image1.JPEG]

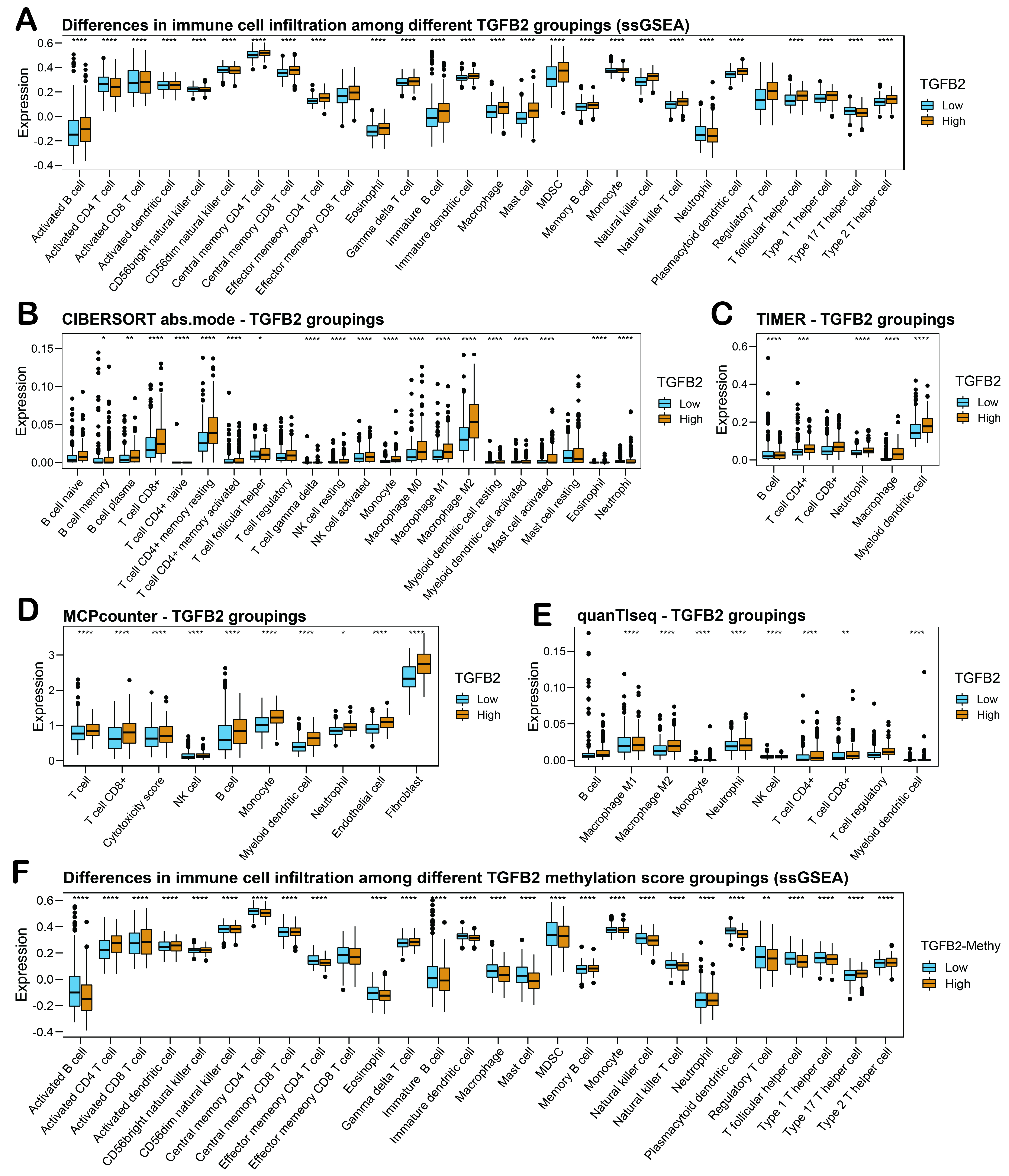

Supplement: Supplementary file 4 [file Image4.JPEG]

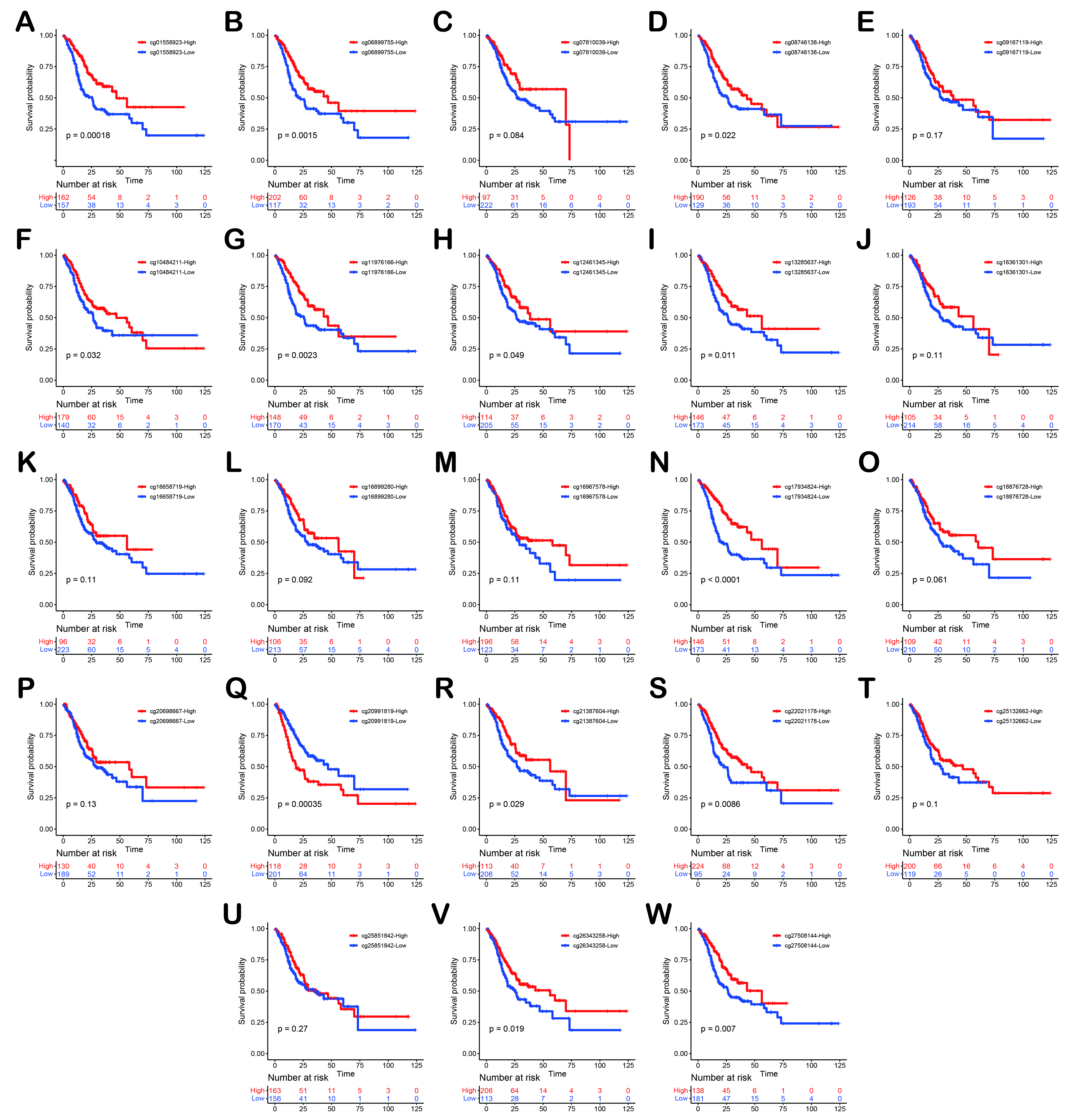

Supplement: Supplementary file 5 [file Image2.JPEG]

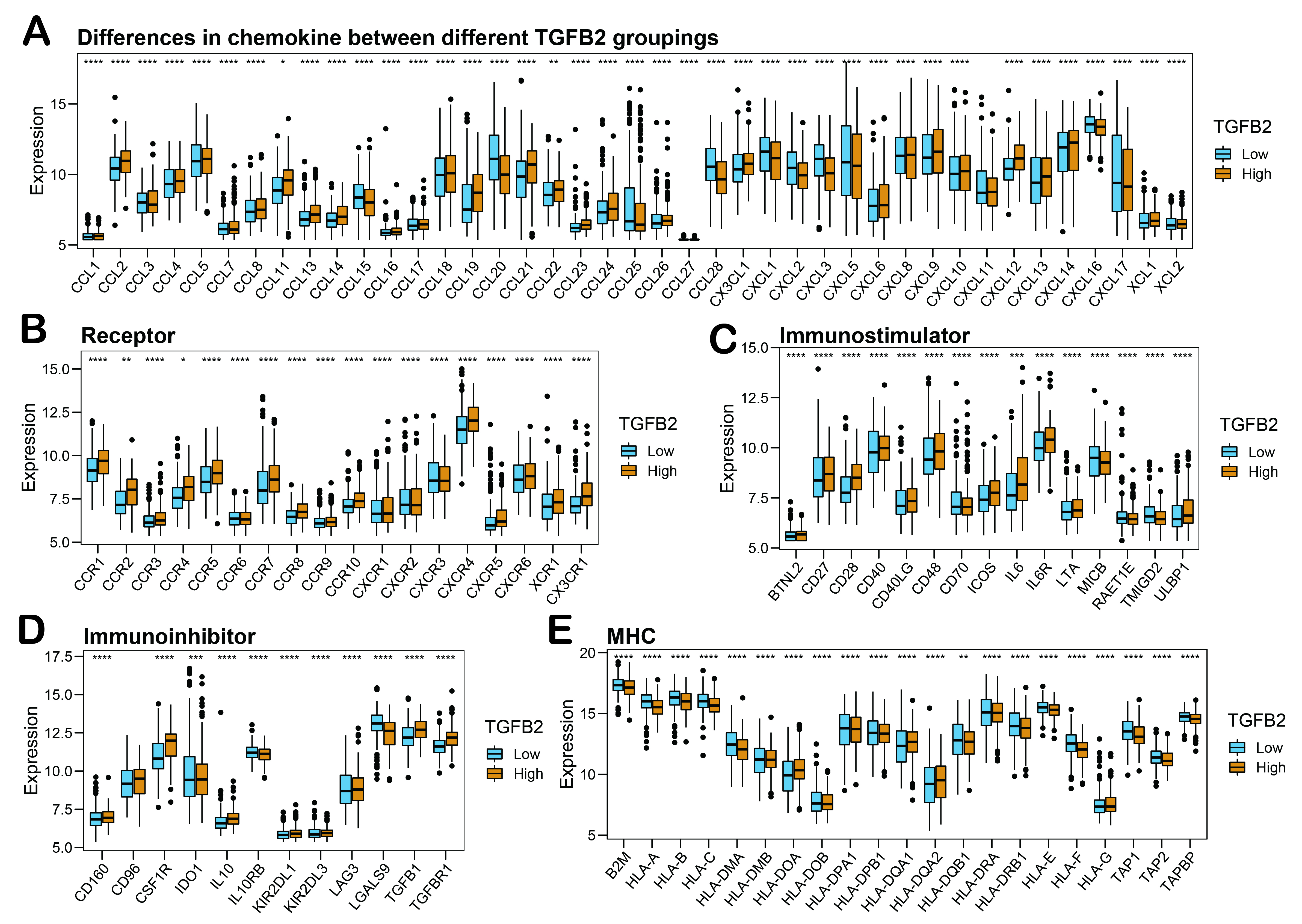

Supplement: Supplementary file 6 [file Image5.JPEG]
