# Supplementary material for: TGFβ2 is a Prognostic Biomarker for Gastric Cancer and is Associated With Methylation and Immunotherapy Responses
Source: Front Genet. 2022 May 10;13:808041. doi: 10.3389/fgene.2022.808041 (PMC9127534; doi:10.3389/fgene.2022.808041)
Supplement: Supplementary file 7 [file Table2.DOCX]

**Supplementary TABLE 2 |**  TGFB2 methylation site details (HumanMethylation450K platform).

| ID | Chromosome | Start | End | feature | cgi |
| --- | --- | --- | --- | --- | --- |
| cg01558923 | chr1 | 218347617 | 218347618 | Body | shore |
| cg06899755 | chr1 | 218346983 | 218346984 | 1stExon | island |
| cg07810039 | chr1 | 218351216 | 218351217 | Body | opensea |
| cg08746138 | chr1 | 218346210 | 218346211 | 1stExon | island |
| cg09167119 | chr1 | 218346234 | 218346235 | 1stExon | island |
| cg10484211 | chr1 | 218374593 | 218374594 | Body | opensea |
| cg11976166 | chr1 | 218346748 | 218346749 | 1stExon | shore |
| cg12461345 | chr1 | 218346223 | 218346224 | 1stExon | island |
| cg13285637 | chr1 | 218347093 | 218347094 | Body | island |
| cg16361301 | chr1 | 218346207 | 218346208 | 1stExon | island |
| cg16658719 | chr1 | 218345621 | 218345622 | TSS1500 | shore |
| cg16899280 | chr1 | 218345237 | 218345238 | TSS1500 | shore |
| cg16967578 | chr1 | 218402095 | 218402096 | Body | opensea |
| cg17934824 | chr1 | 218347450 | 218347451 | Body | shore |
| cg18876728 | chr1 | 218383753 | 218383754 | Body | opensea |
| cg20698667 | chr1 | 218349983 | 218349984 | Body | shelf |
| cg20991819 | chr1 | 218444427 | 218444428 | 3'UTR | opensea |
| cg21387604 | chr1 | 218345126 | 218345127 | TSS1500 | shore |
| cg22021178 | chr1 | 218347126 | 218347127 | Body | island |
| cg25132662 | chr1 | 218364201 | 218364202 | Body | opensea |
| cg25851842 | chr1 | 218345890 | 218345891 | TSS200 | island |
| cg26343258 | chr1 | 218345333 | 218345334 | TSS1500 | shore |
| cg27508144 | chr1 | 218347447 | 218347448 | Body | shore |

Cgi, CpG island.
