# Supplementary material for: TGFβ2 is a Prognostic Biomarker for Gastric Cancer and is Associated With Methylation and Immunotherapy Responses
Source: Front Genet. 2022 May 10;13:808041. doi: 10.3389/fgene.2022.808041 (PMC9127534; doi:10.3389/fgene.2022.808041)
Supplement: Supplementary file 8 [file Table3.DOCX]

**Supplementary TABLE 3 |** The correlation between TGFB2 expression and different immune cell marker genes (TIMER).

| **Description** | **Gene markers** | **STAD** | | | | | |
| --- | --- | --- | --- | --- | --- | --- | --- |
|  |  | **None** | |  | | **Purity** | |
|  |  | **Cor** | ***P*** |  | | **Cor** | ***P*** |
| CD8+ T cell | CD8A  CD8B | 0.169  0.15 | *****  **** | | 0.174  0.161 | | *****  **** |
| T cell (general) | CD3D  CD3E  CD2 | 0.082  0.109  0.16 | 0.097  ***  **** | | 0.082  0.108  0.169 | | 0.109  ***  ***** |
| B cell | CD19  CD79A | 0.153  0.149 | ****  **** | | 0.132  0.129 | | ***  *** |
| Monocyte | CD86  CD115 (CSF1R) | 0.242  0.389 | *****  ***** | | 0.252  0.384 | | *****  ***** |
| TAM | CCL2  CD68  IL10 | 0.337  0.153  0.338 | *****  ****  ***** | | 0.351  0.159  0.359 | | *****  ****  ***** |
| M1 Macrophage | INOS (ISYNA1)  IRF5  COX2(PTGS2) | 0.265  0.263  0.384 | *****  *****  ***** | | 0.285  0.274  0.350 | | *****  *****  ***** |
| M2 Macrophage | CD163  VSIG4  MS4A4A | 0.333  0.331  0.322 | *****  *****  ***** | | 0.334  0.359  0.334 | | *****  *****  ***** |
| Neutrophils | CD66b (CEACAM8)  CD11b (ITGAM)  CCR7 | 0.04  0.342  0.256 | 0.413  *****  ***** | | 0.021  0.352  0.260 | | 0.689  *****  ***** |
| Natural killer cell | KIR2DL1  KIR2DL3  KIR2DL4  KIR3DL1  KIR3DL2  KIR3DL3  KIR2DS4 | 0.105  0.072  -0.09  0.107  0.04  -0.103  0.057 | ***  0.146  0.067  ***  0.416  ***  0.247 | | 0.101  0.058  -0.1  0.103  0.045  -0.109  0.04 | | ***  0.256  0.052  ***  0.381  ***  0.433 |
| Dendritic cell | HLA-DPB1  HLA-DQB1  HLA-DRA  HLA-DPA1  BDCA-1(CD1C)  BDCA-4(NRP1)  CD11c (ITGAX) | 0.087  -0.049  0.021  0.063  0.308  0.568  0.283 | 0.077  0.316  0.664  0.198  *****  1  ***** | | 0.089  -0.061  0.023  0.066  0.323  0.567  0.280 | | 0.084  0.236  0.652  0.199  *****  *****  ***** |
| Treg | FOXP3  CCR8  STAT5B | 0.142  0.291  0.465 | ****  *****  1 | | 0.136  0.297  0.458 | | ****  *****  ***** |

*(Continued)*

**Supplementary TABLE 3 |** Continued

| **Description** | **Gene markers** | **STAD** | | | | | |
| --- | --- | --- | --- | --- | --- | --- | --- |
|  |  | **None** | |  | | **Purity** | |
|  |  | **Cor** | ***P*** |  | | **Cor** | ***P*** |
| Th1 | T-bet (TBX21)  STAT4  STAT1  IFN-γ (IFNG)  TNF-α (TNF) | 0.136  0.283  0.119  -0.027  0.135 | ****  *****  ***  0.585  **** | | 0.145  0.274  0.109  -0.023  0.137 | | ****  *****  ***  0.654  **** |
| Th2 | GATA3  STAT6  STAT5A  IL13 | 0.282  0.134  0.305  0.091 | *****  ****  *****  0.64 | | 0.296  0.136  0.31  0.1 | | *****  ****  *****  0.528 |
| Tfh | BCL6  IL21 | 0.459  0.061 | *****  0.241 | | 0.443  0.066 | | *****  0.2 |
| Th17 | STAT3  IL17A | 0.411  -0.069 | 0  0.163 | | 0.398  -0.071 | | *****  0.165 |
| T cell exhaustion | PD-1 (PDCD1)  CTLA4  LAG3  TIM-3 (HAVCR2)  GZMB | 0.135  0.177  0.073  0.217  -0.03 | ****  *****  0.135  *****  0.541 | | 0.148  0.189  0.07  0.226  -0.04 | | ****  *****  0.173  *****  0.433 |

*STAD, stomach adenocarcinoma; TAM, tumor-associated macrophage; Th, T helper cell; Tfh, Follicular helper T cell; Treg, regulatory T cell; Cor, R value of Spearman’s correlation; None, correlation without adjustment. Purity, correlation adjusted by purity. *P < 0.05; **P < 0.01; ***P < 0.001.*
