# Supplementary material for: TGFβ2 is a Prognostic Biomarker for Gastric Cancer and is Associated With Methylation and Immunotherapy Responses
Source: Front Genet. 2022 May 10;13:808041. doi: 10.3389/fgene.2022.808041 (PMC9127534; doi:10.3389/fgene.2022.808041)
Supplement: Supplementary file 9 [file Table4.DOCX]

**Supplementary TABLE 4 |** The relationship between TGFB2 methylation score and clinicopathological factors in STAD.

| Clinical features | Total | TGFB2 methylation score(%) | | *P*-value |
| --- | --- | --- | --- | --- |
|  |  | score-Low | score-High (%) |  |
| Age  <60  ≥60 | 105  228 | 59(35.3)  108(64.7) | 46(27.7)  120(72.3) | 0.135 |
| Gender  Female  Male | 118  219 | 62(36.7)  107(63.3) | 56(33.3)  112(66.7) | 0.519 |
| TNM stage  Ⅰ  Ⅱ  Ⅲ  Ⅳ | 45  109  149  31 | 19(11.4)  59(35.3)  75(44.9)  14(8.4) | 26(15.6)  50(29.9)  74(44.3)  17(10.2) | 0.546 |
| T stage  T1  T2  T3  T4 | 17  67  160  93 | 4(2.4)  38(22.5)  86(50.9)  41(24.3) | 13(7.7)  29(17.3)  74(44)  52(31) | **0.043** |
| N stage  N0  N1  N2  N3 | 103  86  72  69 | 50(30.3)  48(29.1)  33(20)  34(20.6) | 53(32.1)  38(23)  39(23.6)  35(21.2) | 0.623 |
| M stage  M0  M1 | 300  19 | 147(92.5)  12(7.5) | 153(95.6)  7(4.4) | 0.231 |
| Histologic Grade  G1  G2  G3 | 8  122  198 | 2(1.2)  56(34.4)  105(64.4) | 6(3.6)  66(40)  93(56.4) | 0.171 |

Bold values indicate *P-value* < 0.05; *STAD*, stomach adenocarcinoma.
